# 3D‐Printed Myocardium‐Specific Structure Enhances Maturation and Therapeutic Efficacy of Engineered Heart Tissue in Myocardial Infarction

**DOI:** 10.1002/advs.202409871

**Published:** 2025-01-22

**Authors:** Yong Wu, Yaning Wang, Miao Xiao, Guangming Zhang, Feixiang Zhang, Mingliang Tang, Wei Lei, Ziyun Jiang, Xiaoyun Li, Huiqi Zhang, Xiaoyi Ren, Yue Xu, Xiaotong Zhao, Chenxu Guo, Hongbo Lan, Zhenya Shen, Jianyi Zhang, Shijun Hu

**Affiliations:** ^1^ Institute for Cardiovascular Science & Department of Cardiovascular Surgery of the First Affiliated Hospital State Key Laboratory of Radiation Medicine and Protection Suzhou Medical College Soochow University Suzhou Jiangsu 215000 China; ^2^ Shandong Engineering Research Center for Additive Manufacturing Qingdao University of Technology Qingdao Shandong 266520 China; ^3^ Co‐innovation Center of Neuroregeneration Nantong University Nantong Jiangsu 226001 China; ^4^ Department of Biomedical Engineering School of Medicine and School of Engineering The University of Alabama at Birmingham Birmingham AL 35233 USA; ^5^ Department of Medicine Division of Cardiovascular Disease School of Medicine The University of Alabama at Birmingham Birmingham AL 35233 USA

**Keywords:** 3D printing, engineered heart tissue, myocardial infarction, myocardium‐specific structure

## Abstract

Despite advancements in engineered heart tissue (EHT), challenges persist in achieving accurate dimensional accuracy of scaffolds and maturing human induced pluripotent stem cell‐derived cardiomyocytes (hiPSC‐CMs), a primary source of functional cardiac cells. Drawing inspiration from cardiac muscle fiber arrangement, a three‐dimensional (3D)‐printed multi‐layered microporous polycaprolactone (PCL) scaffold is created with interlayer angles set at 45° to replicate the precise structure of native cardiac tissue. Compared with the control group and 90° PCL scaffolds, the 45° PCL scaffolds exhibited superior biocompatibility for cell culture and improved hiPSC‐CM maturation in calcium handling. RNA sequencing demonstrated that the 45° PCL scaffold promotes the mature phenotype in hiPSC‐CMs by upregulating ion channel genes. Using the 45° PCL scaffold, a multi‐cellular EHT is successfully constructed, incorporating human cardiomyocytes, endothelial cells, and mesenchymal stem cells. These complex EHTs significantly enhanced hiPSC‐CM engraftment in vivo, attenuated ventricular remodeling, and improved cardiac function in mouse myocardial infarction. In summary, the myocardium‐specific structured EHT developed in this study represents a promising advancement in cardiovascular regenerative medicine.

## Graphical abstract

Human iPSC‐derived engineered heart tissue (EHT) mimicking the natural arrangement of myocardial fibers is developed by culturing hiPSC‐derived cardiomyocytes (hiPSC‐CMs) along with supporting cells on a 3D‐printed, multi‐layered polycaprolactone (PCL) scaffold with 45° interlayer angles. These EHTs significantly enhanced hiPSC‐CM maturation and improved cardiac function in mice with myocardial infarction (MI).

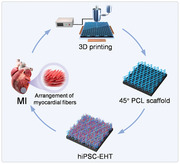

## 1 Introduction

Human induced pluripotent stem cell‐derived cardiomyocytes (hiPSC‐CMs) have become essential tools for cardiovascular research and the treatment of heart disease.^[^ ^1^ ^]^ However, their application in disease modeling and therapy is significantly hindered by their immature state.^[^ ^2^ ^]^ Engineered heart tissue (EHT) represents a promising solution, combining cells and scaffolds to create a tissue structure akin to natural myocardium. Studies have shown that EHT can simulate the human heart environment in vitro, enhance the maturation of hiPSC‐CMs, and serve as a model for heart disease, offering new avenues for treatment and drug screening.^[^ ^3^ ^]^ To better simulate the heart's structure, chamber‐specific EHTs were developed, which have the electrophysiological characteristics of the atria/ventricles.^[^ ^4^ ^]^ Despite these advancements, current EHT models fail to accurately replicate the human heart's structural characteristics. Therefore, developing an EHT model that closely mimics the human heart's structure is essential.

The heart's architecture is defined by multiple layers of myocardial fibers, each arranged at specific angles.^[^ ^5^ ^]^ These geometric features within the myocardial fiber layer guide the cardiomyocytes to align in parallel at a certain angle, facilitating the rapid conduction of action potentials, which is crucial for cardiac tissue maturation and enhancing the heart's contractility.^[^ ^6^ ^]^ Current 3D culture environments lack these geometric cues and thus fail to produce mature myocardial tissue similar to the natural heart structure. Micro‐nano manufacturing technology enables the design of topological structures at various scales to mature cardiomyocytes.^[^ ^7^ ^]^ For instance, grooves at the micro‐ or nanoscale can encourage parallel alignment of cardiomyocytes, enhancing the contractile function of cardiac tissue.^[^ ^8^ ^]^ In addition, geometric shapes also influence cardiomyocyte maturation. Cultivating cardiomyocytes in rectangular shapes that match the dimensions of adult cardiomyocytes can create aligned, uniaxially contracted myocardial bundles, which drive maturation in contractile function, calcium handling, and electrophysiology.^[^ ^9^ ^]^ These topological structures only simulate a single myocardial layer, lack a proper 3D configuration, and cannot accurately replicate the layered characteristics of the human heart.

Three‐demensional (3D) printing technology enables the creation of complex cardiac tissue structures, providing an ideal scaffold for cardiomyocyte growth, optimizing cell arrangement, and thus enhancing cardiomyocyte maturation.^[^ ^10^ ^]^ These 3D scaffolds provide self‐supporting frameworks for cardiomyocytes, allowing them to self‐organize into cardiac tissue or chamber‐like formations.^[^ ^11^ ^]^ Recent research has developed various hydrogel inks to print cardiac tissue models, from ring‐like structures to human ventricular cavities, which can be cultured long‐term and respond to drug stimulation.^[^ ^12^ ^]^ 3D‐printed ventricular models exhibit biomimetic anisotropic electrophysiological and contractile properties, reproducing the heart's periodic contraction.^[^ ^13^ ^]^ However, these models primarily replicate the heart's overall shape and lack the intricate multi‐layered structure of cardiac tissue. While studies indicate that 3D‐printed highly arranged multi‐layer structures can promote cardiomyocyte structural maturation, designing 3D microstructures with optimal pore size, fiber diameter, spacing, and precise inter‐layer angles to support cardiomyocytes remains a significant challenge.^[^ ^14^ ^]^ In our previous study, we employed Electric Field Drive (EFD) technology to create highly ordered 3D microstructures specifically tailored to the dimensions of cardiomyocytes.^[^ ^15^ ^]^ EFD‐based microscale 3D printing offers enhanced electric field stability, which is crucial for constructing multi‐layered 3D scaffolds replicating the heart's distinctive structural features.

**Figure 1:**
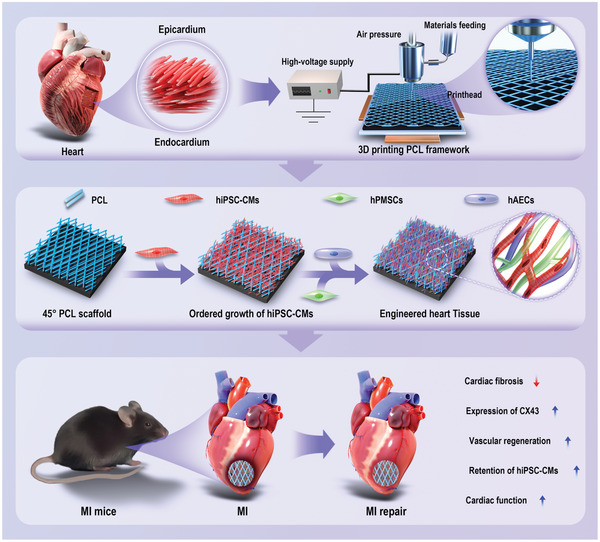
The schematic diagram for constructing engineered cardiac tissue and its application for treating mouse myocardial infarction. Based on the arrangement of pig myocardial fibers, multilayer 45° PCL scaffolds were manufactured using 3D printing. Engineering heart tissue (EHT) was then constructed by culturing hiPSC‐CMs, hAECs, and hPMSCs onto the 3D scaffolds, which promoted the maturation of hiPSC‐CMs. These EHTs can also be applied as a patch to treat myocardial infarction, effectively restoring cardiac function, reducing fibrosis, and promoting angiogenesis in myocardial infarction mouse models.

In this study, we leveraged the in vivo arrangement of myocardial fibers to design and fabricate a multi‐layered 3D microporous polycaprolactone (PCL) scaffold with interlayer angles of 45° using EFD‐based microscale 3D printing technology. Compared with the control group and the 90° PCL scaffold, the 45° PCL scaffold proved more suitable for both the in vitro culture and maturation of hiPSC‐CMs. RNA sequencing analysis demonstrated alterations in extracellular matrix‐related genes and ion channel genes in cardiomyocytes cultured in the 45° PCL 3D scaffold. Using this method, we further constructed an engineered heart tissue with multiple cell types, including cardiomyocytes, endothelial cells, and mesenchymal stem cells, and confirmed its capacity to repair myocardial infarction in mice (**Figure** **1**).

## 2 Results

### 2.1 Preparation and Characterization of 3D PCL Scaffolds

**Figure 2:**
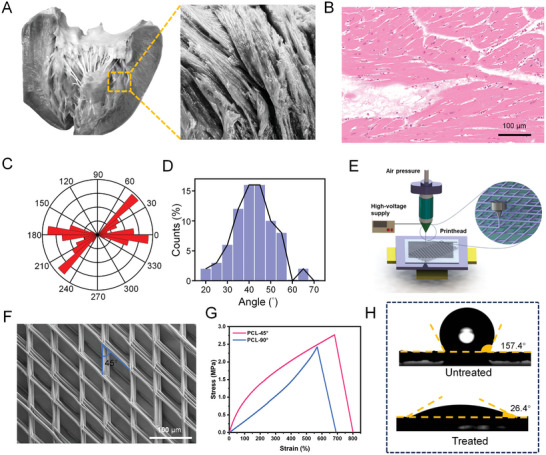
Preparation and characterization of PCL porous scaffolds. A) Anatomy of the pig heart and myocardial fiber arrangement. B) HE staining of the transverse section of the pig myocardial tissue. C) Statistics of the length of different pig heart muscle fibers angles. Data were acquired from 8831 sarcomeric points. D) Representative histogram of different angles of the pig heart muscle fibers. E) 3D printing model of the PCL scaffold. F) SEM image of the 3D‐printed 45° PCL scaffold. G) Mechanical testing of the PCL scaffold (45° and 90°). H) Hydrophilicity test of the PCL scaffold before and after alcohol gradient treatment.

In this study, we analyzed pig heart tissue to determine the arrangement of myocardial muscle fibers. As shown in **Figure** **2A**, the muscle fibers are arranged in a spiral cross at specific angles. HE staining of the transverse section of heart tissue further indicated that the muscle fibers were arranged at various angles (Figure 2B,C), most commonly between 30° and 55° (Figure 2D). Therefore, we selected 45° as the specific arrangement angle for the 3D PCL scaffolds, with 90° as the comparative control.

PCL scaffolds were prepared using EFD jet deposition micro‐nano 3D printing technology (Figure 2E). By leveraging the shrinkage effect of the electric field on the fibers, they were deposited at a microscale onto the substrate, producing a highly porous structure with small pores conducive to cell growth. The prepared PCL scaffolds featured a line width of 20 µm, a rectangular pore shape, and dimensions of 60 µm by 80 µm. SEM images confirmed the uniform pore size of the prepared biological scaffolds (Figure 2F). The thermal properties of PCL were analyzed using differential scanning calorimetry (DSC) and thermal gravimetric analysis (TGA), revealing a melting point of 67.5 °C (Figure S1A, Supporting Information) and a degradation phase between 327.2 °C and 427.3 °C (Figure S1B, Supporting Information).

Mechanical testing showed that the scaffold had a tensile strength of 2.77 MPa (Figure 2G), making it suitable for in vivo use. After gradient alcohol hydrophilic treatment, the contact angle between the water droplet and the PCL scaffold decreased from 157.4° to 26.4° (Figure 2H), indicating that the scaffold became hydrophilic and thus suitable for subsequent cell experiments.

### 2.2 3D PCL Scaffolds Oriented at 45° Facilitated the Maturation of hiPSC‐CMs

**Figure 3:**
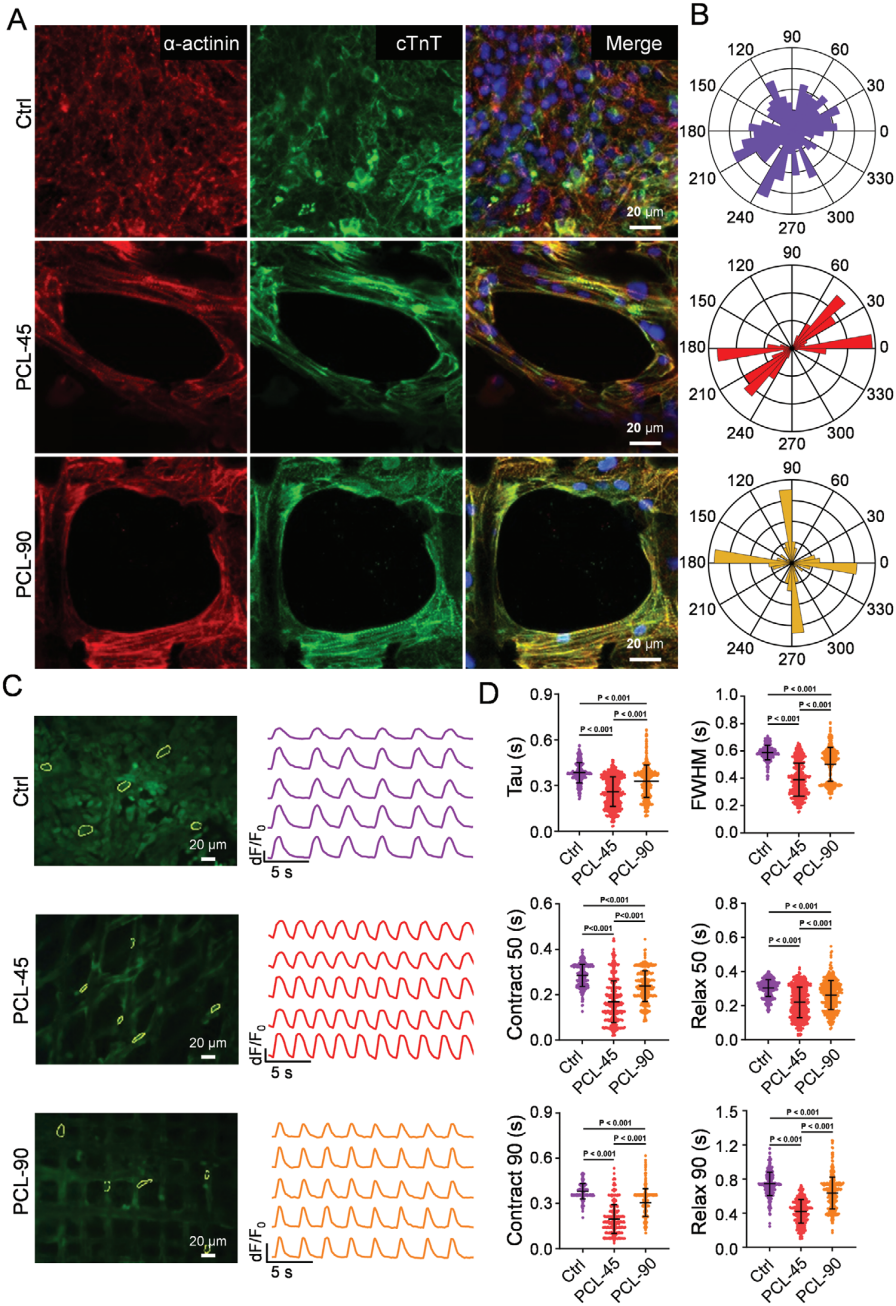
Characterization of hiPSC‐CMs cultured on the PCL scaffolds with different arrangement angles. A) Immunofluorescence staining of cTnT and α‐actinin on hiPSC‐CMs on hiPSC‐CMs cultured on cell culture slides (Ctrl), 90° PCL scaffolds, and 45° PCL scaffolds. B) Representative histogram of polarity in hiPSC‐CMs in different groups. Data were acquired from 22 143 sarcomeric points in the Ctrl group, 8062 sarcomeric points in the 45° PCL scaffold, and 14 975 sarcomeric points in the 90° PCL scaffold. C) Representative calcium transient traces and curves of hiPSC‐CMs. D) Calcium transient parameters (tau, FWHM, Contract 50, Relax 50, Contract 90, Relax 90) of hiPSC‐CMs in different groups. Data were acquired from 375 transits in the Ctrl group, 475 transits in the 45° PCL scaffold, and 407 transits in the 90° PCL scaffold. Data are presented as mean ± SD, *p*‐values were calculated using one‐way ANOVA with Bonferroni post hoc test.

The purified hiPSC‐CMs were cultured on coverslips (Control group, Ctrl), as well as on 45° (PCL‐45) and 90° (PCL‐90) PCL scaffolds, respectively. Immunofluorescence staining for α‐actinin and cTnT revealed that hiPSC‐CMs aligned with the direction of the scaffold, showing an orderly arrangement. In contrast, the orientation of hiPSC‐CMs on the Ctrl was random and non‐directional (**Figure** **3**A). The alignment of CMs on the 45° PCL scaffolds was more pronounced than those on the Ctrl and 90° scaffolds (Figure 3B)

Additionally, we measured calcium transients in CMs across different groups using Fluo4‐AM (Figure 3C). Statistical analysis indicated that CMs cultured on the 45° PCL scaffolds demonstrated better calcium handling capacity compared to the other groups, evidenced by a reduction in calcium transient parameters, including time constant (Tau), full width at half maximum (FWHM), Contract 90, Relax 90, Contract 50, and Relax 50 (Figure 3D). These findings suggest that the 45° PCL scaffolds were more conducive to the growth and maturation of hiPSC‐CMs than the Ctrl and the 90° PCL scaffolds. Therefore, the 45° PCL scaffolds exhibited a fiber arrangement akin to the natural heart's, enhancing the CMs' calcium handling capacity and maturity.

### 2.3 RNA Sequencing Analysis of hiPSC‐CMs Cultured on the 45° PCL Scaffolds

**Figure 4:**
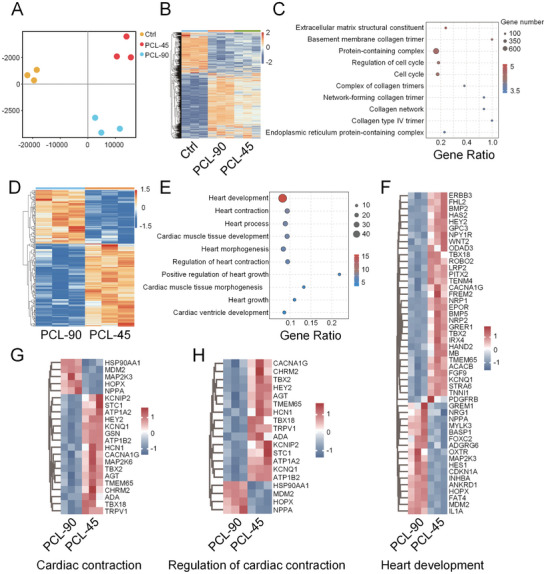
Transcriptome analyses of hiPSC‐CMs on PCL scaffold at different angles. A) Principal component analysis of all samples’ global gene expression profiles (n = 3 per group). B) Heatmap of differentially expressed genes in profile 6 and profile 1 (FDR < 0.05 and fold change (FC) > 1.5). C) GO analysis of differentially expressed genes in profile 6 and profile 1. D) Heatmap of differentially expressed genes in hiPSC‐CMs cultured on PCL scaffolds with different angles (FDR < 0.05 and fold change (FC) > 1.5). E) GO analysis of differentially expressed genes in hiPSC‐CMs cultured on 90° and 45° PCL scaffolds. F‐H) Heatmap of differential expressed genes enriched in three GO terms: heart development, cardiac contraction, and regulation of cardiac contraction (FDR < 0.05 and fold change (FC) > 1.5).

To analyze the potential mechanisms by which 3D PCL scaffolds affect cardiomyocyte maturation, we performed RNA‐seq on hiPSC‐CMs from the ctrl, PCL‐90, and PCL‐45 groups. Principal component analysis (PCA) revealed significant differences in mRNA expression profiles among the three groups (**Figure** **4A**). We performed trend analysis on the differentially expressed genes in the Ctrl, PCL‐90, and PCL‐45 groups. The expression trends of all genes can be divided into 8 profiles, among which profile 6 and profile 1 are significant (Figure S2A, Supporting Information). The genes in profile 6 and profile 1 have similar expression levels in the PCL‐90 and PCL‐45 groups and are upregulated or down‐regulated relative to the Ctrl group (Figure 4B). Gene Ontology (GO) analysis indicated that these genes are associated with extracellular matrix (ECM) function (Figure 4C). Compared to the Ctrl group, the PCL‐90 and PCL‐45 scaffolds mainly affected the expression of ECM‐related genes (Figure 4D). These suggest that 3D PCL scaffolds promote the maturation of hiPSC‐CMs mainly by regulating ECM function and composition.

Next, we wanted to know how the angle further affects the maturation of hiPSC‐CMs. There were 801 differentially expressed genes in the PCL‐45 and PCL‐90 groups (Figure S2B, Supporting Information). GO analysis showed that these genes were significantly enriched in system and multi‐cellular organ development (Figure S2C, Supporting Information). We performed GO analysis again on the genes in these two GO terms, focusing on the effects of PCL‐45 on heart development and function. Compared with PCL‐90, PCL‐45 mainly affected several GO terms, such as heart development, cardiac contraction, and myocardial tissue development (Figure 4E). Compared with PCL‐90, the expression levels of major genes related to heart development in the PCL‐45 group, such as *BMP2*, *WNT2*, *TBX18*, and *TBX2*, were significantly increased (Figure 4F). Since PCL‐45 can promote calcium maturation of hiPSC‐CMs (Figure 3), calcium ions are related to cardiomyocyte contraction. We focused on the expression of genes in the cardiac contraction and regulation of cardiac contraction GO terms in PCL‐90 and PCL‐45. In these two GO terms, ion channel‐related genes, such as *KCNIP1*, *ATP1A2*, *KCNQ1*, *ATP1B2*, *CACNA1G*, and *KCNIP2*, were significantly upregulated in PCL‐45 (Figure 4G,H). These results suggest that compared with 90°, 45° promotes the maturation of hiPSC‐CMs mainly by regulating the expression of cardiac development and ion channel genes.

### 2.4 Construction and Maturation of the 3D‐Engineered Heart Tissue

**Figure 5:**
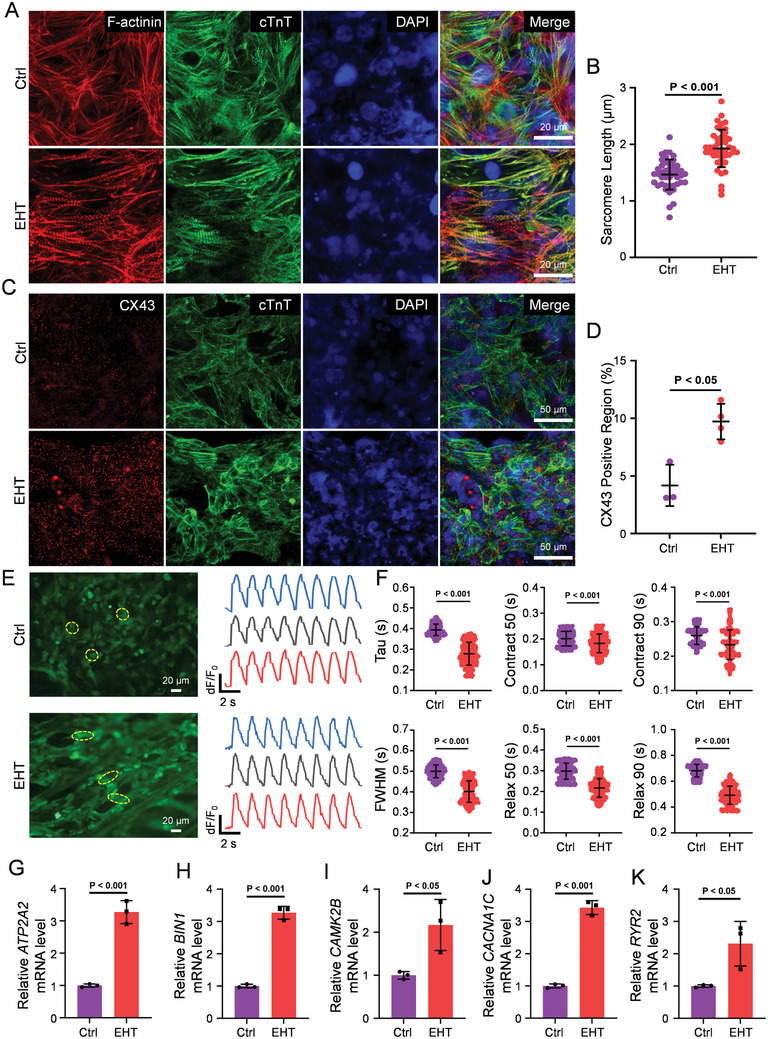
Evaluation of the 45° PCL scaffold‐based 3D engineered heart tissue with multi‐cellular lineage. A) Immunofluorescence staining of cTnT and F‐actinin on the Ctrl and the EHT. B) Statistics of sarcomere length of hiPSC‐CMs. Data were acquired from 45 sarcomeres points in both Ctrl and EHT groups. C) Immunofluorescence staining of C X 43 and cTnT on hiPSC‐CMs in the Ctrl and the EHT groups. D) Statistics of gap junction protein C X 43 expression in the Ctrl (n = 3) and EHT (n = 4) groups. E) Immunofluorescence imaging of cardiomyocytes using the calcium fluorescent dye Fluo‐4 AM and simultaneous calcium transients obtained from the labeled cardiomyocytes. Data were acquired from 455 transits in Ctrl group and 455 transits in EHT. F) Comparison of calcium transient parameters: relative tau, FWHM, Contract 50, Relax 50, Contract 90, and Relax 90. G–K) Relative expression of *ATP2A2*, *BIN1*, *CAMK2B*, *CACNA1C*, and *RYR2* in EHT. Data are presented as mean ± SD, *p*‐values were calculated using Student's t‐test.

To better simulate the physiological state of the heart, we further constructed a more complex EHT using the 45° PCL scaffold through the supplementation of endothelial cells and interstitial cells. In this study, hiPSC‐CMs, hAECs, and hPMSCs were co‐cultured in a 45° PCL scaffold at a ratio of 6:1:1. Specifically, cTnT and F‐actin immunofluorescence staining of the EHT showed that the cells grew well within the scaffold and were orderly arranged along the scaffold fibers. Further analysis of their growth direction indicated that the distribution direction of hiPSC‐CMs in the 45° PCL scaffold was approximately 45°, more orderly than that in the control group (**Figure** **5A**; Figure S3A,B, Supporting Information). Compared to the control group, the sarcomeres of hiPSC‐CMs in the EHT group were neatly aligned, and the sarcomere length was longer, reaching 1.93 ± 0.33 µm (Figure 5B). Besides, cTnT and CX43 cell immunofluorescence co‐staining (Figure 5C) showed that the expression of CX43 in the EHT group was significantly increased compared with the control group (Figure 5D).

Calcium imaging of hiPSC‐CMs was performed using the calcium fluorescent dye Fluo‐4 AM (Figure 5E). Subsequently, ImageJ and Python software were utilized to analyze the acquired images, and the results demonstrated that the values of Tau, FWHM, Contract 50, Relax 50, Contract 90, and Relax 90 were all reduced compared to the control group (Figure 5F), indicating an enhanced calcium handling capacity of hiPSC‐CMs in the EHT. In addition, qPCR analysis of the engineered heart tissue revealed that the mRNA expression levels of *ATP2A2*, *BIN1*, *CAMK2B*, *CACNA1C*, and *RYR2* were upregulated compared to the control group (Figure 5G–K). These findings suggest that EHT's calcium handling ability is improved.

We further evaluated the drug responsiveness of EHTs through calcium transient analysis. Following the inhibition of spontaneous calcium transients with verapamil, we measured the amplitude of caffeine‐induced calcium transients in cardiomyocytes. Compared to the controls, hiPSC‐CMs in EHTs exhibited a stronger response to caffeine (Figure S3C, Supporting Information), indicating enhanced calcium release from the sarcoplasmic reticulum (SR). These suggest that hiPSC‐CMs in EHTs are more mature and well‐suited for drug testing or screening applications.

Therefore, we successfully constructed the 3D EHT by cultivating human cardiomyocytes, endothelial cells, and mesenchymal stem cells on the 45° PCL scaffolds, where hiPSC‐CMs grew orderly and exhibited more mature phenotypes.

### 2.5 The EHT Promoted the Recovery of Cardiac Function after MI

Before conducting in vivo therapy research, we first evaluated the biocompatibility of PCL by transplanting PCL scaffolds onto normal mouse hearts. Cardiac function, assessed 30 days post‐transplantation, showed no significant differences between the PCL and Sham groups (Figure S4A–C, Supporting Information). Meanwhile, HE staining of tissue sections revealed no obvious pathological changes in the heart, liver, spleen, lungs, and kidneys of mice from the PCL group (Figure S4D, Supporting Information). These findings suggest that PCL did not induce significant biological toxicity in the mice.

**Figure 6:**
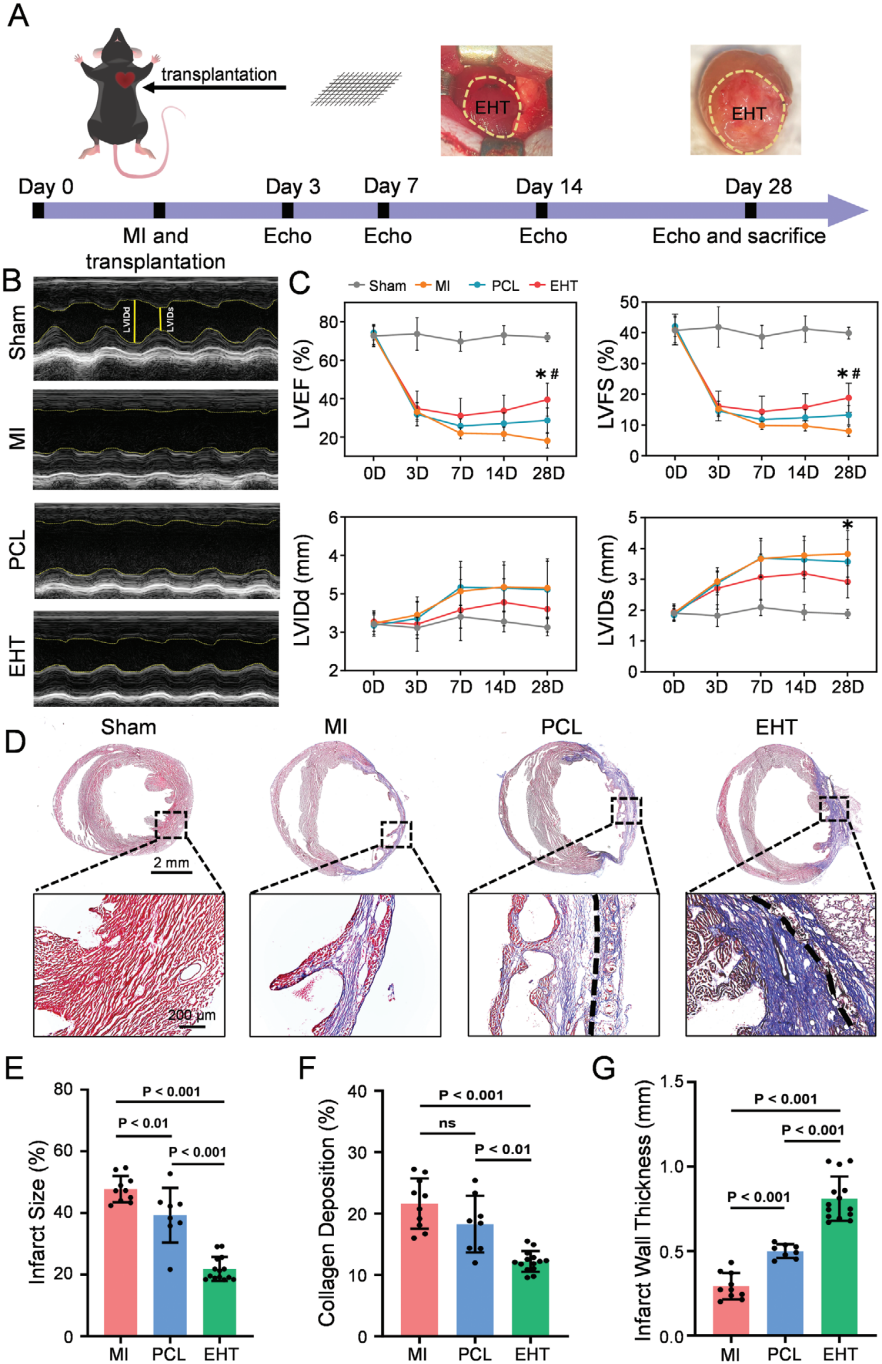
Improvement of cardiac function in mouse MI after EHT transplantation. A) Timeline of myocardial infarction construction and echocardiography detection in mice. B) Representative echocardiogram images of Sham, MI, PCL, and EHT groups. C) Statistical analysis of left ventricular ejection fraction (LVEF), left ventricular fractional shortening (LVFS), left ventricular internal diameter in diastole (LVIDd), left ventricular internal dimension in systole (LVIDs) at different time points. For Sham, MI, PCL, and EHT groups, n = 12, 12, 13, and 14, respectively. *: Statistically significant differences between EHT and MI (*p* < 0.01), #: Statistically significant differences between EHT and PCL (*p* < 0.01). D) Representative Masson trichrome staining images of tissue sections after 28 days in Sham, MI, PCL, and EHT groups. E ‐G) Statistical analysis of infarct size (E), collagen deposition (F), and wall thickness in the infarct area (G) in MI (n = 10), PCL (n = 8), and EHT (n = 14) groups. Data are presented as mean ± SD, *p*‐values were calculated using one‐way ANOVA with Bonferroni post hoc test.

To investigate the therapeutic efficacy of EHT patches on MI, we transplanted EHT into the infarct area of MI mice. We assessed cardiac function on days 3, 7, 14, and 28 post‐surgery using small animal echocardiography (**Figure** **6A**). The echocardiography results on day 28 revealed that the anterior wall of the left ventricle in the MI group exhibited a straight line with minimal to no contraction. In contrast, the anterior wall of the left ventricle in the EHT group demonstrated good contractility with a peak value (Figure 6B). Cardiac functions, including LVEF, LVFS, LVIDd, and LVIDs, were analyzed via echocardiography. The MI group showed significantly lower cardiac function compared to the other groups, with an LVEF of 18.1% ± 3.6%, LVFS of 8.8% ± 1.3%, LVIDd of 4.2 ± 0.7 mm, and LVIDs of 3.8 ± 0.7 mm at day 28. In contrast, the EHT group exhibited significantly improved cardiac function, with an LVEF of 39.5% ± 8.25%, LVFS of 18.5% ± 3.0%, LVIDd of 3.6 ± 0.6 mm, and LVIDs of 2.9 ± 0.5 mm at day 28, indicating better therapeutic effects in vivo. Meanwhile, the LVEF value in the PCL group increased gradually, reaching 28.7% ± 6.2% at day 28, which was still lower than that of the EHT group (Figure 6C).

Masson's trichrome staining (Figure 6D) and statistical analysis (Figure 6E–G) showed that the myocardial tissue of mice in the MI group had more collagen deposition than the Sham group. The infarction wall became thinner, and the ventricular chamber was dilated, indicating significant ventricular remodeling. However, the PCL group had reduced the infarct size and collagen deposition with no significant difference in collagen deposition, while both were significantly reduced in the EHT group. Statistical analysis indicated that the infarct wall thickness was 0.29 ± 0.07 mm in the MI group, 0.50 ± 0.04 mm in the PCL group, and 0.81 ± 0.13 mm in the EHT group. These results demonstrate that the EHT significantly improves cardiac function and decreases ventricular remodeling after MI.

### 2.6 The EHT Patches Increased Gap Junction and Angiogenesis in the MI Area

**Figure 7:**
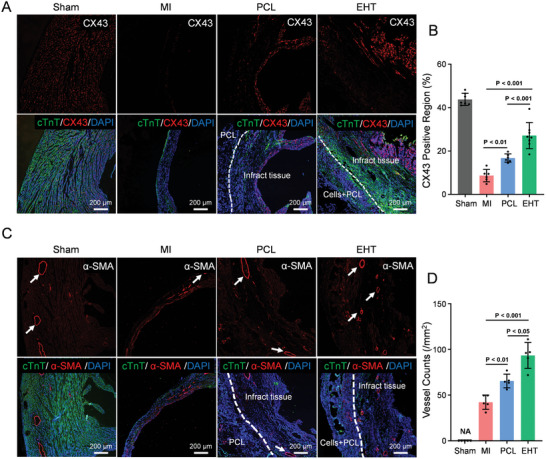
Improvement of gap junction and angiogenesis in mouse MI with EHT treatment. A) Immunofluorescence staining of cTnT and CX43 in mouse infarct area of Sham, MI, PCL, and EHT groups. B) Statistical analysis of CX43 expression in the infarct area of mice in Sham (n  =  7), MI (n  =  6), PCL (n  =  6) and EHT (n  =  8) groups. C) Immunofluorescence staining of cTnT and α‐SMA in mouse infarct area of Sham, MI, PCL, and EHT groups. D) Statistical analysis of vascular density in the infarct area of MI, PCL and EHT groups (n  =  5 per group). Data for vascular density was not available (NA) in the Sham group since no infarct area was present. Data are presented as mean ± SD. *p*‐values were calculated using one‐way ANOVA with Bonferroni post hoc test.

To evaluate the integration of EHT patches with the host myocardium's surface, heart tissue samples in the EHT group were stained with an antibody specifically recognizing the human cTnT protein. Many hiPSC‐CMs survived in the EHT group (Figure S5, Supporting Information). Tissue samples were further co‐stained with an antibody recognizing both human and mouse cTnT and an antibody recognizing the gap junction protein CX43, critical for myocardial electrical signal conduction (**Figure** **7A**). Compared to the MI and the PCL groups, the EHT group exhibited higher expression levels of CX43 (Figure 7B).

Angiogenesis is vital for restoring blood supply to the infarct area and improving heart function. Immunofluorescence staining with cTnT/α‐SMA (Figure 7C) showed a significant increase in microvessels within the infarct area in the EHT group compared to the MI and PCL groups (Figure 7D). These results suggest that the EHT enhances gap junction formation and promotes angiogenesis in the myocardial infarction area.

## 3 Discussion

Heart tissue is a highly organized structure where cardiomyocytes are connected end‐to‐end and unidirectionally. These cardiomyocytes form myocardial fibers oriented at specific angles within the heart.^[^ ^16^ ^]^ Most studies have focused on the relationship between topological structure and myocardial maturation, the level of individual cardiomyocytes, or single‐layer myocardial fibers. By considering the overall cardiac structure and the morphological characteristics of cardiomyocytes, and based on anatomical studies of the pig heart,^[^ ^17^ ^]^ we developed a 45° 3D PCL porous scaffold that mimics the myocardial fiber arrangement. Our findings demonstrate that the 45° scaffold significantly promotes the maturation of hiPSC‐CMs in EHT, leading to a marked improvement in cardiac function when transplanted into the infarcted myocardium in mice. This indicates that engineered scaffolds replicating myocardial fiber structures are more suitable for future clinical applications.

The heart's extracellular matrix (ECM) provides anchoring and fixation support for cardiomyocyte arrangement, enabling electrical coupling and pulse propagation between cells.^[^ ^18^ ^]^ After birth, to adapt to the increase in cardiac volume and contractility, the protein composition and molecular structure of the ECM change, rapidly developing into a highly aligned, interconnected, and intertwined network.^[^ ^19^ ^]^ When the myocardial structure is damaged, it leads to uneven electrophysiological distribution, resulting in arrhythmias.^[^ ^20^ ^]^ The PCL scaffold we constructed has a continuous, interconnected 3D‐ordered structure, providing a natural adult‐like ECM environment for the growth of hiPSC‐CMs.

Although the natural myocardial fibers in pigs exhibit a range of orientations, predominantly between 35° and 55°, incorporating multi‐layers with different alignments in the 3D PCL scaffold results in very small pores, making effective cell seeding challenging. Therefore, based on the predominant fiber orientation observed in the pig heart, we selected a 45° alignment to construct the 3D‐ordered PCL scaffolds. Our study demonstrates that, compared with a 90° alignment, the 45° orientation can promote the maturation of calcium handling in hiPSC‐CMs. RNA‐seq analysis further indicates that the 45° orientation significantly promotes the expression of ion channel genes related to action potentials and calcium handling. These suggest that the 45° PCL scaffold is more in line with the structural characteristics of the myocardium and is conducive to the electrophysiological activity of hiPSC‐CMs.

Single‐cell RNA sequencing of adult hearts reveals that ventricular tissue contained 49.2% cardiomyocytes and 7.8% endothelial cells, with a cardiomyocyte‐to‐endothelial cell ratio of about 6:1.^[^ ^21^ ^]^ Endothelial cells interact with cardiomyocytes through direct cell‐to‐cell contact and paracrine effects.^[^ ^22^ ^]^ Previous studies have shown that ECs can promote cardiomyocyte maturation.^[^ ^20^, ^23^ ^]^ Despite their low abundance, MSCs have been shown to promote angiogenesis and the expression of cardiomyocyte‐specific markers by secreting soluble factors such as VEGF, FGF, and angiopoietin.^[^ ^24^ ^]^ Therefore, to better mimic the cellular composition of the heart and MI treatment, we constructed a multi‐cellular hybrid EHT by co‐culturing hiPSC‐CMs, hAECs, and hPMSCs on the PCL 45° scaffold. Consistent with existing research results, adding hAECs and hPMSCs further promotes the maturation of hiPSC‐CMs and brings them closer to adult cardiomyocytes' functional state.

Given that many myocardial cells die following MI, the primary goal of MI therapy is to replenish myocardial cells in the infarct area. However, cell injection therapy often leads to cell loss, significantly reducing its therapeutic efficacy. The EHT can improve cell retention rates.^[^ ^14^ ^]^ Additionally, MI can lead to ventricular remodeling, collagen deposition, wall thinning, and ventricular dilation.^[^ ^25^ ^]^ Studies have shown scaffolds rely on mechanical properties to support the MI area and prevent ventricular rupture.^[^ ^26^ ^]^ Cell‐free PCL scaffolds also demonstrated efficacy in treating MI in this study. As myocardial infarction progresses, fibrosis hinders cardiac electrical propagation.^[^ ^27^ ^]^ The EHT prepared with the 45° PCL scaffold in this study significantly improved the hiPSC‐CMs retention rates, enhanced angiogenesis in the infarct area, and provided mechanical support during the early stages of MI to prevent ventricular rupture. Furthermore, it maintained CX43 expression in the infarcted area, which is beneficial for myocardial electrical conduction.

Due to its high mechanical strength,^[^ ^28^ ^]^ PCL is commonly used in cardiac applications as a fiber scaffold, significantly lowering its tensile modulus.^[^ ^29^ ^]^ In our study, the tensile strength of PCL scaffolds was reduced to 2–3 MPa. While this remains higher than that of healthy heart tissue (10‐15 kPa) and infarcted heart tissue (50 kPa),^[^ ^30^ ^]^ studies have shown that incorporating non‐contractile materials that are up to 200% stiffer than the natural heart tissue can offer several benefits, such as enhanced structural support and reduced pressure on surrounding tissues.^[^ ^31^ ^]^ Additionally, patches with Young's modulus in the range of tens of kilopascals to several megapascals have been found to promote cardiac function recovery after myocardial infarction.^[^ ^32^ ^]^ Moreover, PCL scaffolds will gradually lose stiffness in the later stages post‐transplantation due to their biodegradability, which occurs over ≈2–3 years. This property, combined with the porous structure of our PCL scaffolds that facilitates cellular growth, represents a promising feature for the long‐term integration of EHTs. However, PCL alone is not conductive, which poses a limitation given the natural electrical conductivity of the myocardium. To address this, developing conductive scaffolds incorporating PCL with conductive materials like MXene and a 45° alignment structure, may further enhance cardiomyocyte maturation and promote effective myocardial injury repair.

In summary, we constructed a 45° multi‐layer 3D scaffold based on the physiological structure of the heart. Compared to other angled scaffolds, this myocardial‐specific 3D scaffold significantly enhanced the structural maturation and calcium handling capacity of hiPSC‐CMs. It closely resembled the myocardial fibers of the native heart. The EHT formed by the co‐culturing of hiPSC‐CMs, hAECs, and hPMSCs further enhances the maturity of hiPSC‐CMs. When transplanted into the infarcted area, the EHT promoted vascular regeneration, enhanced cell‐cell connection, and improved the cardiac function of mice with myocardial infarction. Our work provides a novel approach to applying engineered heart tissue in cardiovascular regenerative medicine.

## 4 Experimental Section

### Preparation and Characterization of the 3D PCL Scaffold

Given its biocompatibility and slow degradation rate, polycaprolactone (PCL) was selected for constructing 3D scaffolds in this study. The study aimed to replicate the arrangement of pig myocardial fibers using Electric Field Drive (EFD) jet deposition, a micro‐nano 3D printing technology. PCL porous and ordered scaffolds were fabricated at angles of 45° and 90° on pre‐treated substrates. The process involved applying a voltage at the nozzle, causing the PCL material to form a Taylor cone at the nozzle's exit, generating a conical jet. Under the influence of an electric field, the PCL material was ejected from the nozzle and printed onto the substrate, forming PCL porous ordered scaffolds at the specified angles. The scaffold structure was initially observed using a S4800 Scanning Electron Microscopy (Hitachi, Japan), and the mechanical properties were measured with an Instron machine (Instron, UK). TGA and DSC were performed using TGA/DSC 3+ Mettler Toledo (Switzerland). The PCL scaffold was then subjected to a gradient treatment of ethanol concentrations (75%, 50%, 25%, and 5%), followed by three washes with sterile double‐distilled water. The scaffold was then coated with Matrigel, and its hydrophilicity was measured using a contact angle meter.

### Cardiomyocyte Differentiation from hiPSCs

The hiPSCs, previously generated by reprogramming of human urine cells, were routinely maintained in Matrigel‐coated culture dishes using PSCeasy E8 medium (Cellapy, China) and passaged with 0.5 mM EDTA (Sigma‐Aldrich, USA) at ≈80% confluence.^[^ ^33^ ^]^ For cardiomyocyte differentiation, hiPSCs at about 85% confluence were cultured in CDM3 medium and treated with 5 µM CHIR‐99021 (Sigma‐Aldrich, USA) and 2 µM C59 (Selleck Chemicals, USA) for 48 h each. The cells were subsequently maintained in a CDM3 medium. Spontaneously beating cardiomyocytes were generally observed on day 8. After passaging, cardiomyocytes were purified in a glucose‐free medium containing lactic acid before being used for subsequent experiments.^[^ ^34^ ^]^

### Construction of Engineered Heart Tissue

Scaffold structures were divided into coverslips (Ctrl), 45°, and 90°. The PCL scaffolds were treated with a gradient of ethanol concentrations (75%, 50%, 25%, and 5%) and then rinsed three times with sterile double‐distilled water. Next, they were soaked in a DMEM/1640 (Gibco, USA) mixed medium for 12 hours, then coated with Matrigel. After purification, hiPSC‐CMs were digested with 0.25% trypsin‐EDTA to form a single‐cell suspension and then seeded onto Matrigel‐coated coverslips, 45° and 90° PCL scaffolds, with 0.6×10^6^ cells per scaffold. To ensure uniform distribution, we first dropped 200 µL of cell suspension onto the PCL scaffold, allowing the suspension to wrap the scaffold completely. After the cells adhered to the scaffold, the EHTs were cultured in the appropriate cell culture medium. The cells were cultured in CDM3 medium, with the medium replaced every other day. They were used for subsequent experiments after 10 days of in vitro culture. For myocardial infarction treatment, 45° scaffolds were selected based on in vitro results, and human aortic endothelial cells (hAECs) and human placenta mesenchymal stem cells (hPMSCs) were added to form a three‐cell mixed culture of engineered heart tissue.

### RNA Sequencing

After hiPSC‐CMs were cultured on coverslips, 45°, and 90° scaffolds for 10 days, total RNA was extracted using the RNA extraction kit (Vazyme, China). RNA integrity was assessed using an Agilent 2100 bioanalyzer, agarose gel electrophoresis, a NanoPhotometer spectrophotometer, and a Qubit 2.0 Fluorometer. RNA sequencing (RNA‐seq) analysis and library construction were then performed. Data quality control, sequence alignment, gene analysis, expression level statistics, gene‐ontology (GO) enrichment analysis, and Kyoto Encyclopedia of Genes and Genomes (KEGG) enrichment analysis were conducted. Statistical significance was set at a fold change greater than 2 and a p‐value less than 0.05. Data analysis was carried out using the transcriptome analysis platform of the Beijing Genomics Institute (BGI).

### Cell and Tissue Immunofluorescence Staining

Cultured cells and tissue sections were fixed with 4% paraformaldehyde (PFA), permeabilized with 0.1% Triton X‐100, and blocked with 5% bovine serum albumin (BSA) at room temperature for 1 hour. Samples were incubated with primary antibodies diluted in PBST: anti‐cTnT (1:200), anti‐α‐Actinin (1:200), anti‐α‐SMA (1:200), and anti‐CX43 (1:200), all from Abcam, UK, overnight at 4 °C or for 2 h at room temperature. After washing in PBST three times for 5 min each, samples were incubated for 1 h at room temperature with complementary secondary antibodies diluted in PBST: Alexa Fluor 555‐labeled goat anti‐rabbit secondary antibody (1:500) and Alexa Fluor 647‐labeled goat anti‐mouse secondary antibody (1:500), both from Life Technologies (USA). After washing in PBST three times for 5 min each, nuclei were counterstained with DAPI (Invitrogen, USA) diluted in PBST for 7 min at room temperature. Following the final wash, samples were sealed with a fluorescence quencher (VectorLabs, USA). Observations were made under an LSM 880 confocal microscope (Zeiss, Germany) using low magnification to observe cell arrangement and high magnification to observe sarcomere arrangement and CX43 expression in hiPSC‐CMs. Images were taken for subsequent analysis.

### Calcium Transient Analysis of hiPSC‐CMs

After 7 days of engineered heart tissue culture, cells were incubated in a culture medium supplemented with 4 µM Fluo‐4 AM (Life Technologies, USA) for 30 min, followed by washing twice with Ringer's solution for 1 min each.^[^ ^35^ ^]^ Calcium transient imaging was conducted using an Olympus IX51 microscope at a magnification of 20× objective, with an acquisition speed of 50 ms per frame,^[^ ^36^ ^]^ for 60 s. ImageJ and Python software were utilized to quantitatively analyze intracellular calcium ion fluorescence intensity. Parameters such as frequency, waveform intensity, and half‐width of the calcium transient were compared under different culture conditions.

### Real‐Time Quantitative PCR (qPCR)

Total RNA was extracted from the engineered heart tissues using Trizol reagent and reverse transcribed into cDNA using reverse transcriptase SuperMix (YEASEN, China). The expression levels of myocardial maturation‐related genes, including *ATP2A2*, *BIN1*, *CAMK2B*, *CACNA1C*, and *RYR2*, were quantified using an SYBR Green Master kit (YEASEN, China). The primers used are listed in Table S1 (Supporting Information).

### Construction of Myocardial Infarction Model

Male C57BL/6 mice (22 g – 25 g) were purchased from the Experimental Animal Center of Soochow University (Jiangsu, China). All animal procedures were approved by the Institutional Animal Care and Use Committee of the First Affiliated Hospital of Soochow University (No. 2023–349) and were conducted in strict accordance with the National Institutes of Health (NIH) guidelines for the care and use of laboratory animals (NIH Publication No. 85‐23 Rev. 1985). The mice were randomly divided into four groups: Sham group, MI group, PCL group (PCL patches in the infarction area post‐surgery), and EHT group (engineered heart tissue patches in the infarction area post‐surgery). Mice were anesthetized via intraperitoneal injection of pentobarbital sodium (50 mg kg^−1^). The hair on the neck and chest was removed using a depilatory cream. Mice were intubated and connected to a ventilator. The thoracic cavity was opened between the third and fourth ribs, and the pericardium was exposed by opening the sternum. The left anterior descending coronary artery (LAD) was permanently ligated with a 6‐0 nylon suture. Successful MI establishment was confirmed by pale heart apex and ST‐segment elevation on the electrocardiogram. In the PCL and EHT groups, patches were transplanted onto the infarct surface and secured with the pericardium. The Sham group underwent the same surgical procedure without LAD ligation. Postoperatively, mice were placed on a 37 °C electric heating blanket to recover and allowed normal food and water intake. To prevent immune rejection, cyclophosphamide (50 mg kg^−1^) was administered intraperitoneally one day before surgery and on days 1, 3, 7, and 14 post‐surgery.

### Echocardiography

Echocardiography was performed on mice using a small animal ultrasound scanner before surgery and at days 3, 7, 14, and 28 post‐surgeries. Mice were anesthetized with isoflurane and secured on a testing platform. The left ventricular long axis was measured, and M‐mode echocardiography was utilized to evaluate the following parameters of ventricular function: left ventricular ejection fraction (LVEF), left ventricular fractional shortening (LVFS), left ventricular internal diameter in diastole (LVIDd), left ventricular internal dimension in systole (LVIDs).

### Masson Staining and HE Staining

Mice in the Sham, MI, PCL, and EHT groups were euthanized at 28 days post‐MI. The hearts were then collected and fixed in 4% (w/v) paraformaldehyde (PFA) before being dehydrated overnight with 30% (w/v) sucrose. The infarcted hearts were embedded in the O.C.T. compound, and 7 µm sections were cut for histological analysis. Masson's trichrome staining was utilized to assess the extent of fibrosis, with the infarct size calculated as the ratio of the perimeter of the blue‐stained area to the total left ventricular perimeter using ImageJ software. The average wall thickness of the infarct area was also measured using the same software. Tissue morphology and angiogenesis in the MI region were observed through hematoxylin and eosin (HE) staining.

### In Vivo Assessment of Biocompatibility

Biocompatibility testing was performed on C57 male mice. PCL scaffolds were transplanted onto the surface of mouse hearts. Cardiac function, including LVEF and LVFS, were assessed 30 days post‐transplantation. Subsequently, the mice were euthanized, and the heart, liver, spleen, lungs, and kidneys were collected to evaluate the toxicity of the PCL scaffold to these organs through HE staining.

### Statistical Analyses

Data are presented as the mean ± standard deviation (SD). At least five samples were tested in each group in vivo experiment and at least three per group were evaluated in the in vitro experiment. The Kolmogorov–Smirnov test was used to assess the normality of the data. Comparisons between the two groups were analyzed using Student's t‐test. Multiple comparison correction analysis was performed using one‐way analysis of variance (ANOVA) with Bonferroni post hoc test. Statistical analyses were performed using GraphPad Prism 9.0. A p‐value of <0.05 indicated statistical significance.

## Conflict of Interest

The authors declare no conflict of interest.

## Author Contributions

Yo.W., Ya.W., M.X., G.Z., and F.Z. contributed equally as co‐first authors. Yo.W., Ya.W., M.X., G.Z., F.Z., and S.H. conceived and designed the experiments. Yo.W., Ya.W., F.Z., X.L., H.Z., X.R., and X.Z. performed the experiments. Yo.W., Ya.W., M.X., Z.J., M.T., W.L., and Y.X. analyzed the data. Yo.W., Ya.W., and S.H. wrote and edited the manuscript. G.Z., C.G., H.L. provided materials and were involved in discussions of the work. J.Z. contributed to project discussion and manuscript revision. Z.S. and S.H. supervised the project. All authors discussed the results and commented on the manuscript.

## Supporting information



Supporting Information

## Data Availability

The data that support the findings of this study are available in the supplementary material of this article.
