# Supplementary material for: 3D‐Printed Myocardium‐Specific Structure Enhances Maturation and Therapeutic Efficacy of Engineered Heart Tissue in Myocardial Infarction
Source: Adv Sci (Weinh). 2025 Jan 22;12(10):2409871. doi: 10.1002/advs.202409871 (PMC11905000; doi:10.1002/advs.202409871)
Supplement: Supplementary file 1 — Supporting Information [file ADVS-12-2409871-s001.docx]

**SUPPLEMENTAL INFORMATION**

**3D-Printed Myocardium-Specific Structure Enhances Maturation and Therapeutic Efficacy of Engineered Heart Tissue in Myocardial Infarction**

Yong Wu^1,*^, Yaning Wang^1,*^, Miao Xiao^1,*^, Guangming Zhang^2,*^, Feixiang Zhang^1,*^, Mingliang Tang^1,3^, Wei Lei^1^, Ziyun Jiang^1^, Xiaoyun Li^1^, Huiqi Zhang^1^, Xiaoyi Ren^1^, Yue Xu^1^, Xiaotong Zhao^1^, Chenxu Guo^2^, Hongbo Lan^2^, Zhenya Shen^1^, Jianyi Zhang^4, 5^, Shijun Hu^1,#^

^1^ Institute for Cardiovascular Science & Department of Cardiovascular Surgery of the First Affiliated Hospital, State Key Laboratory of Radiation Medicine and Protection, Suzhou Medical College, Soochow University, Suzhou, Jiangsu 215000, China; ^2^ Shandong Engineering Research Center for Additive Manufacturing, Qingdao University of Technology, Qingdao, Shandong 266520, China; ^3^ Co-innovation Center of Neuroregeneration, Nantong University, Nantong, Jiangsu 226001, China; ^4^ Department of Biomedical Engineering, School of Medicine and School of Engineering, The University of Alabama at Birmingham, AL 35233, United States; ^5^ Department of Medicine, Division of Cardiovascular Disease, School of Medicine, The University of Alabama at Birmingham, AL 35233, United States.

^*^ co-first authors

^#^ Correspondence to: Shijun Hu, Email: [shijunhu@suda.edu.cn](mailto:shijunhu@suda.edu.cn)

**
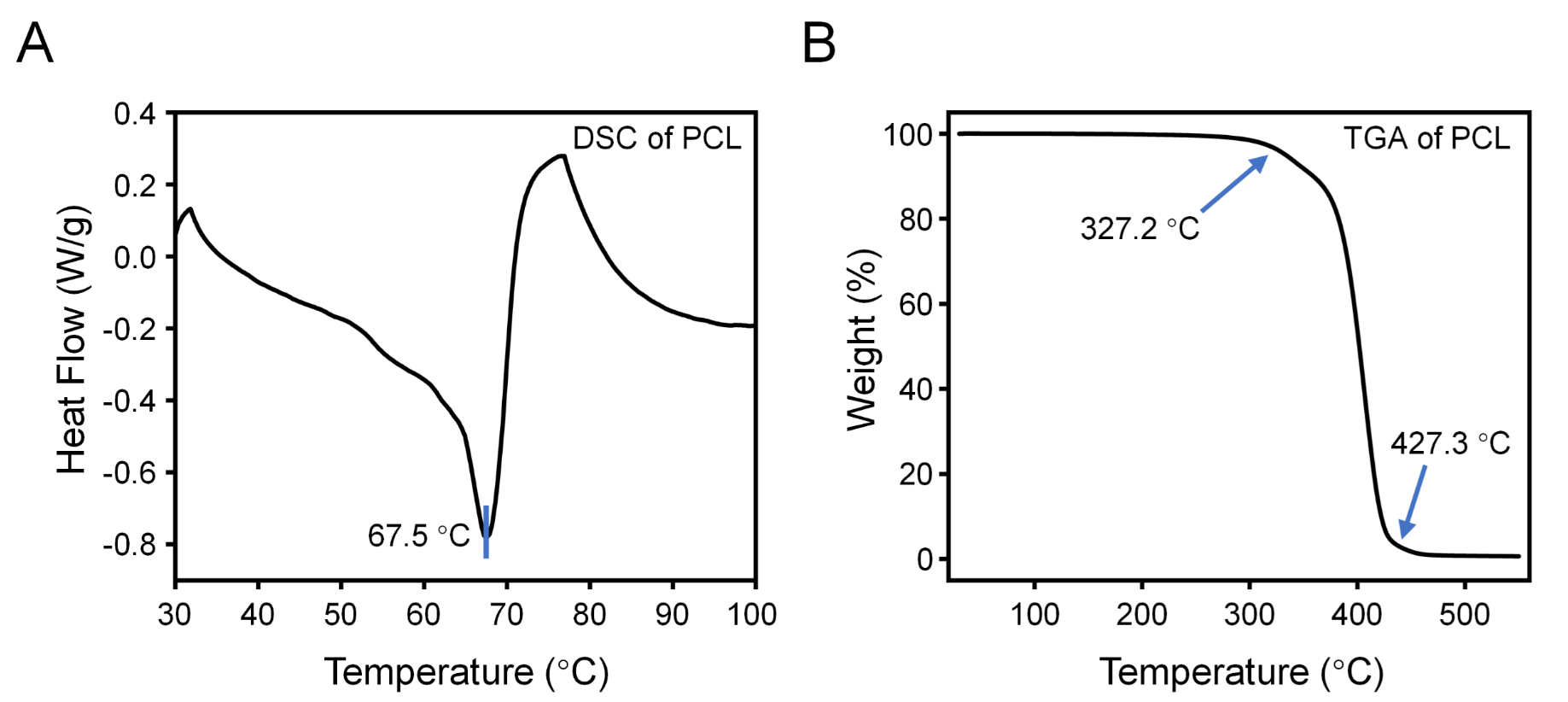
**

**Figure S1.** The thermal properties of the PCL. A) The DSC curves for PCL have a melting point of 67.5 °C. B) The TGA curves for PCL. PCL shows a degradation step from 327.2 °C to 427.3 °C.

**
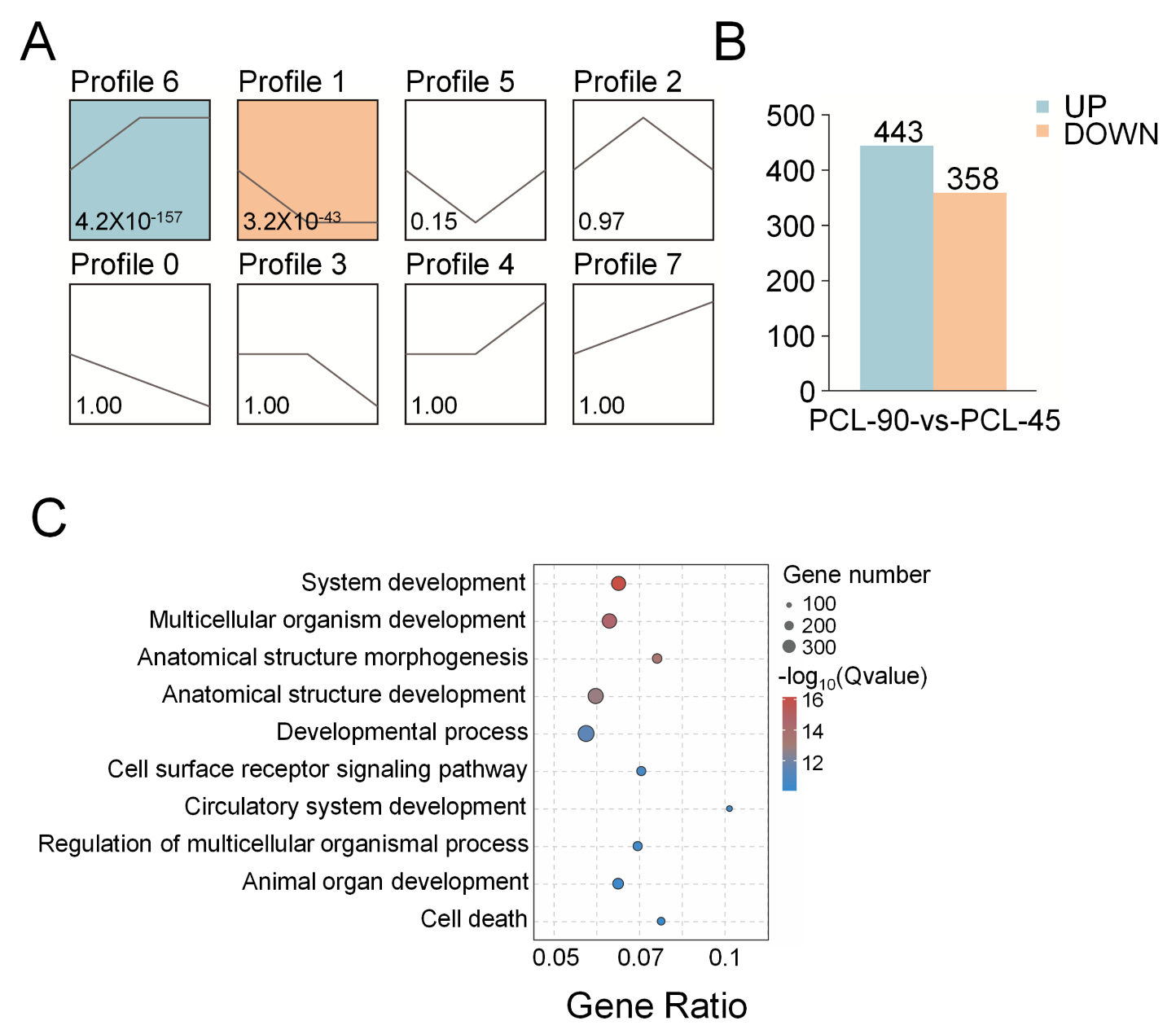
**

**Figure S2.** Analysis of differentially expressed genes in hiPSC-CMs cultured on Ctrl, 90°and 45° PCL scaffolds. A) Expression trends of differentially expressed genes in Ctrl, PCL-90, and PCL-45 groups (P value < 0.05 and fold change (FC) > 1.5). B) Number of differentially expressed genes in hiPSC-CMs cultured on 90° and 45° PCL scaffolds. C) GO analysis of differentially expressed genes in PCL-90 and PCL-45 groups.

**
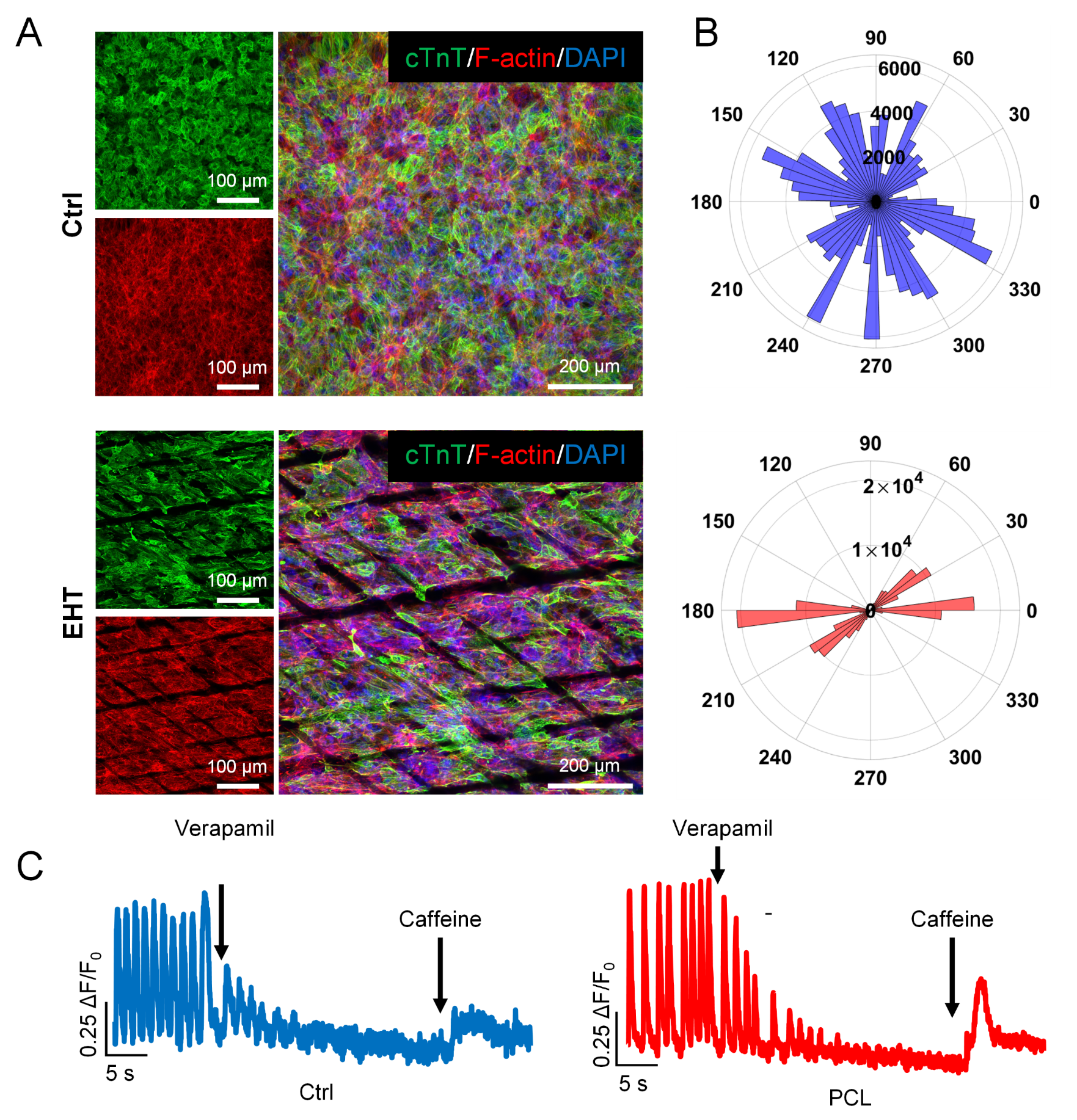
**

**Figure S3.** Orientation distribution and drug responsiveness of hiPSC-CMs on 45° PCL scaffolds. A) Growth of hiPSC-CMs, hAECs, and hPMSCs on coverslips and 45° PCL scaffolds, and immunofluorescence staining of cTnT and F-actin. B) Representative polarity histograms of hiPSC-CMs cultured on coverslips and 45° PCL scaffolds. C) Representative calcium traces with verapamil and caffeine treatment in the control and PCL groups, and hiPSC-CMs on PCL showed a strong response to caffeine.


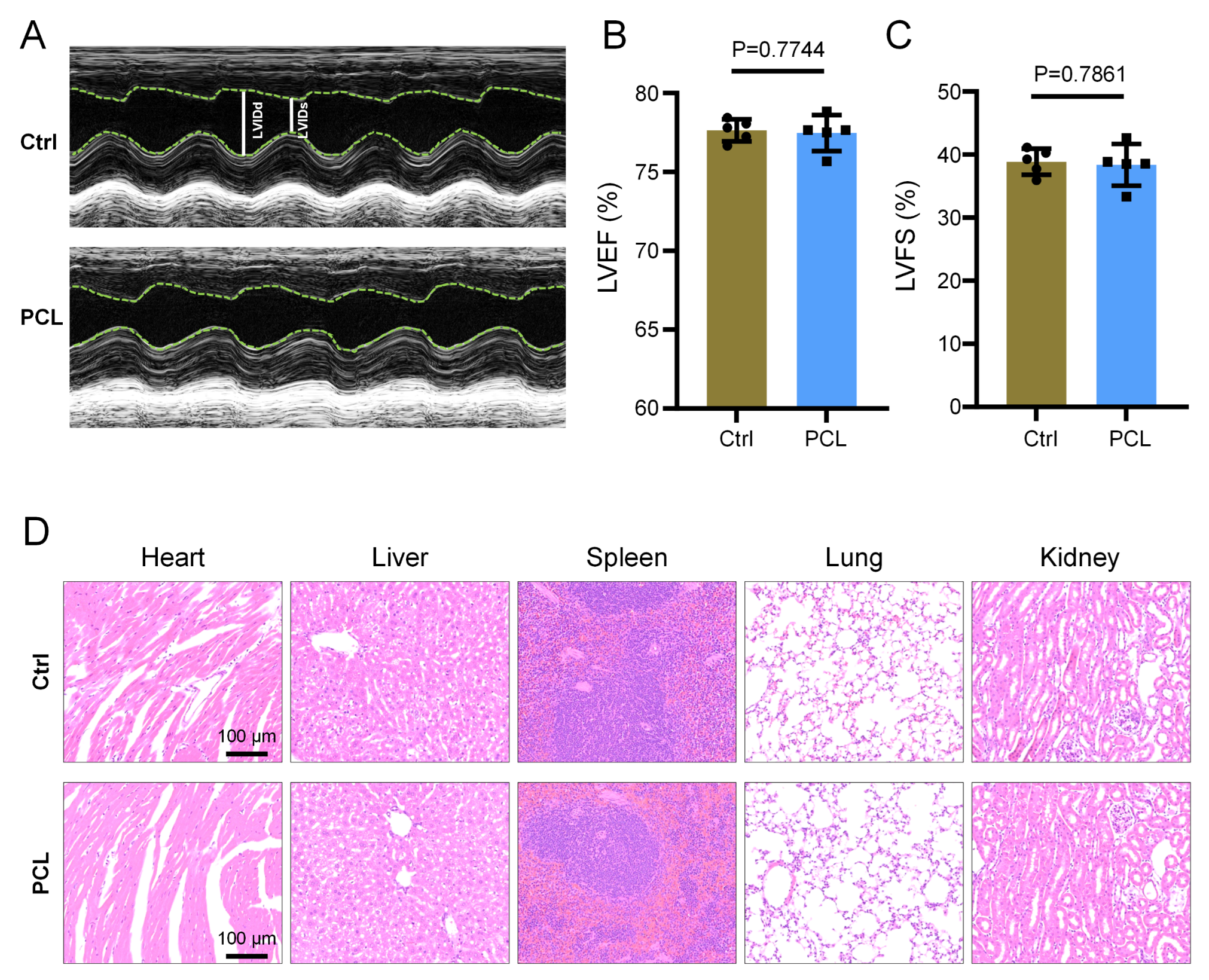


**Figure S4.** Biocompatibility analyses of PCL in mice at day 30 post-transplantation of PCL scaffold onto normal mouse hearts. A) Representative echocardiogram images of Ctrl and PCL groups. B) Statistical analysis of left ventricular ejection fraction (LVEF) at day 30 in Ctrl (n = 5) and PCL (n = 5) groups. C) Statistical analysis of left ventricular fractional shortening (LVFS) at day 30 in Ctrl (n = 5) and PCL (n = 5) groups. D) HE staining of sections from heart, liver, spleen, lungs, and kidneys in Ctrl and PCL groups, scar bars: 100 μm. Data are presented as mean ± SD, P-values were calculated using Student’s t-test.


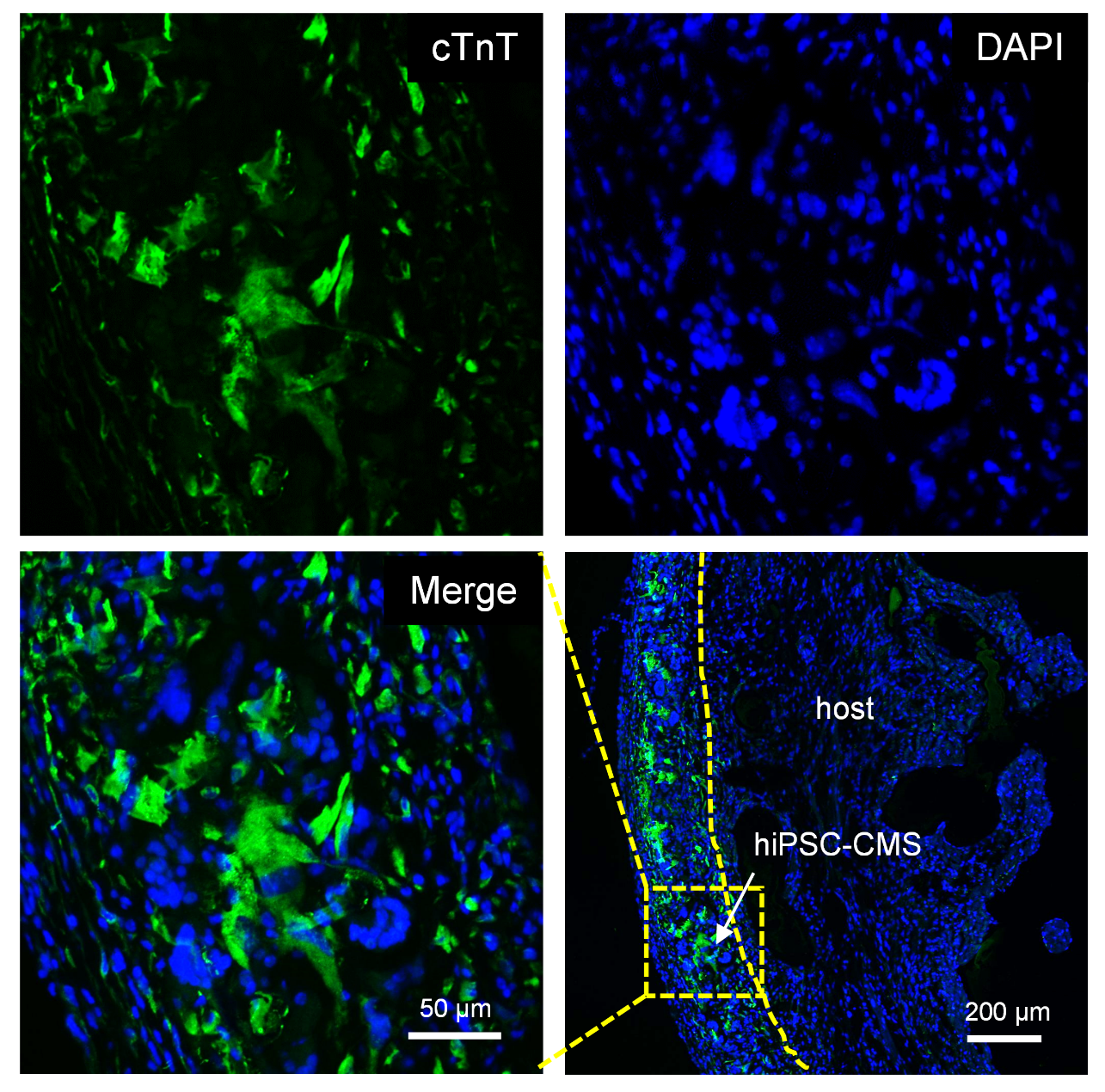


**Figure S5.** Representative images showing cTnT immunostaining in mouse MI heart at day 28 after EHT transplantation. The hiPSC-CMs (green) were identified by immunofluorescent staining with human specific cTnT antibody.

**Supplemental Table S1. Primers used for qPCR in the study.**

| **Targets** | **Primer sequence (5'-3')** |
| --- | --- |
| *18S* | F-GTAACCCGTTGAACCCCATT |
|  | R- CCATCCAATCGGTAGTAGCG |
| *ATP2A2* | F- ATGGGGCTCCAACGAGTTAC |
|  | R- CCATCCAATCGGTAGTAGCG |
| *BIM* | F-ATGAGGCAAACAAGATCGCAG |
|  | R-CGTGACTTGATGTCGGGGAA |
| *CAMK2B* | F-GCACACCAGGCTACCTGTC |
|  | R-CATACGCCTCTTTGCGAAGG |
| *CACNA1C* | F- CATGCTCACGGTGTTCCA |
|  | R- TCCTACGGCATCATTGACC |
| *RYR2* | F- TCCGGAAACAGTATGAAGACCA |
|  | R- CACACAACGCTGGCAATTCA |
| F, forward; R, reverse. | |

**Supplemental Table S2. Antibodies used in the study.**

| **Antibody name** | **Company, Catalog Number.** | **Dilution** |
| --- | --- | --- |
| cTnT | Abcam, ab45932 | 1:200 for IF |
| cTnT | Abcam, ab8295 | 1:200 for IF |
| α-actinin | Abcam, ab9465 | 1:200 for IF |
| CX43 | Abcam, ab11370 | 1:200 for IF |
| α-SMA | Proteintech, 67735-1-Ig | 1:200 for IF |
| F-actinin | Abcam, ab176759 | 1:200 for IF |
| Immunofluorescence (IF) | | |
